# Architecture of the herpesvirus genome-packaging complex and implications for DNA translocation

**DOI:** 10.1007/s13238-020-00710-0

**Published:** 2020-04-23

**Authors:** Yunxiang Yang, Pan Yang, Nan Wang, Zhonghao Chen, Dan Su, Z. Hong Zhou, Zihe Rao, Xiangxi Wang

**Affiliations:** 1 CAS Key Laboratory of Infection and Immunity, National Laboratory of Macromolecules, Institute of Biophysics, Chinese Academy of Sciences, Beijing, 100101 China; 2 State Key Laboratory of Biotherapy, West China Hospital, Collaborative Innovation Center for Biotherapy, Sichuan University, Chengdu, 610041 China; 3 Department of Microbiology, Immunology, and Molecular Genetics, University of California, Los Angeles, Los Angeles, CA 90095 USA; 4 Laboratory of Structural Biology, School of Medicine, Tsinghua University, Beijing, 100084 China; 5 State Key Laboratory of Medicinal Chemical Biology and College of Life Science, Nankai University, Tianjin, 300353 China

**Keywords:** dsDNA virus, genome packaging, viral maturation, terminase complex, drug target

## Abstract

Genome packaging is a fundamental process in a viral life cycle and a prime target of antiviral drugs. Herpesviruses use an ATP-driven packaging motor/terminase complex to translocate and cleave concatemeric dsDNA into procapsids but its molecular architecture and mechanism are unknown. We report atomic structures of a herpesvirus hexameric terminase complex in both the apo and ADP•BeF3-bound states. Each subunit of the hexameric ring comprises three components—the ATPase/terminase pUL15 and two regulator/fixer proteins, pUL28 and pUL33—unlike bacteriophage terminases. Distal to the nuclease domains, six ATPase domains form a central channel with conserved basic-patches conducive to DNA binding and trans-acting arginine fingers are essential to ATP hydrolysis and sequential DNA translocation. Rearrangement of the nuclease domains mediated by regulatory domains converts DNA translocation mode to cleavage mode. Our structures favor a sequential revolution model for DNA translocation and suggest mechanisms for concerted domain rearrangements leading to DNA cleavage.

**Electronic supplementary material:**

The online version of this article (10.1007/s13238-020-00710-0) contains supplementary material, which is available to authorized users.

## Introduction

**Figure 1:**
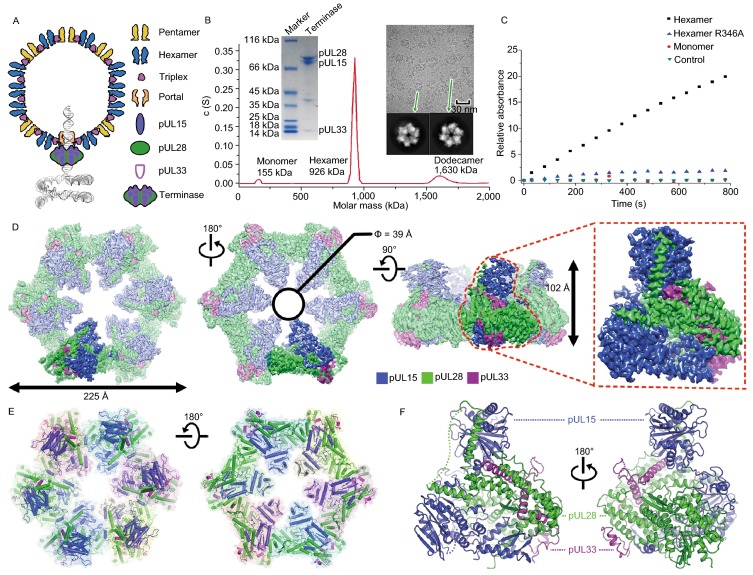
**Characterization and overall structure of the hexameric terminase assembly**. (A) Model for herpesvirus procapsid during DNA packaging. (B) Characterization of the terminase complex analyzed by analytical ultracentrifugation, SDS-PAGE and electron microscopy. (C) Representative curves of the MESG-based assays to measure the ATPase activities of wild-type and R346A mutant hexameric terminase rings and wild-type monomer terminase complex. (D) CryoEM map of the hexameric terminase assembly. The inset shows the blocked-based reconstruction for one terminase complex, which consists of pUL15 (blue), pUL28 (green) and pUL33 (magenta). (E and F) Atomic models for the hexameric terminase assembly and the terminase complex. Color scheme is the same as Fig. 1D

Viruses use one of two main strategies to package genomes: either assembling a capsid around the genome (e.g., HIV) or packaging the genome into a preformed capsid (Sun et al., 2010; Dai and Zhou, 2018). Most large double-stranded DNA (dsDNA) viruses use the latter strategy. Examples include herpesviruses and most bacteriophages, both of which replicate their DNA as head-to-tail concatemers containing multiple copies of the genome that must be cleaved to generate unit-length viral genomes (Sun et al., 2008). This is achieved using a “terminase” complex, which, in bacteriophages comprises two proteins, termed Large (TerL) and Small (TerS) terminases; but in herpesviruses contains three separate components—pUL15, pUL28 and pUL33—in hitherto unknown oligomeric forms. The terminase complex recognizes a specific sequence or structure on the concatemeric DNA and cuts it to produce the free end where packaging is initiated (Mettenleiter et al., 2009). Acting as the packaging motor, the terminase complex then hydrolyzes ATP molecules to translocate negatively charged DNA into a space-limited procapsid through a dodecameric portal (Chen, 2020; Nan Wang, et al. 2020) (Fig. 1A). In line with its essential roles in viral maturation, the genome-packaging terminase complexes from herpesviruses are excellent targets for various Food and Drug Administration (FDA)-approved anti-viral drugs by blocking the formation of infectious virions (Bogner, 2002; Melendez and Razonable, 2015).

Although advances in our understanding of this process in bacteriophages have been made (Sun et al., 2007; Sun et al., 2008; Hilbert et al., 2015), the stoichiometry of TerL and TerS of the bacteriophage terminase complex and their assembly are poorly understood in bacteriophages. Difficulty in isolation of an intact terminase complex has hampered biochemical analysis and delineation of the structural basis for viral genome packaging in dsDNA viruses. Additionally, there remain important unanswered questions and even debates regarding the compositions, architectures, and evolution of genome-packaging complexes and the mechanisms by which these motor complexes function (Guo et al., 1998, 2013; Sun et al., 2010; Heming et al., 2017). Here we reported the atomic structures of a herpesvirus hexameric terminase complex in both the apo and ADP•BeF3 bound states at 3.5 Å and 3.6 Å. The architecture of the hexameric terminase assembly reveals structural features necessary for sequential revolution DNA translocation and concerted DNA cleavage, addressing the puzzle of how dsDNA viruses efficiently package their genome.

## Results

### Characterization and overall structure of the hexameric terminase assembly

We co-expressed the three components of the herpesvirus terminase complex, pUL15, pUL28 and pUL33, using a baculovirus-based expression system. Surprisingly, three types of terminase assemblies, monomeric, hexameric and dodecameric, were observed in the expressed protein samples. Interestingly, the proteins predominantly assembled in a hexameric form (Fig. 1B). Cryo electron microscopy (cryoEM) of the hexameric form revealed a compact assembly (Fig. 1B). Biochemical assays showed that the hexameric form hydrolyzed ATP more rapidly when compared to the monomeric form, which exhibited low activity (Fig. 1C). To elucidate the structural mechanism of viral DNA packaging, we determined the cryoEM structures of the hexameric and dodecameric terminase assemblies in the apo state at 3.9 Å and 4.6 Å resolution, with C6 and D6 symmetry respectively; and those of the hexameric and dodecameric terminase bound with an ATP mimic, ADP•berylliumtrifluoride (ADP•BeF3, a nonhydrolyzable ATP analog to mimic ATP in physiological conditions (Ren et al., 2019)) at 4.3 Å and 4.6 Å resolution, respectively (Figs. 1D, S1 and S2). By using the block-reconstruction method (Yuan et al., 2018; Zhu et al., 2018a), we were able to improve the resolution for the structures of the hexameric and dodecameric terminases to 3.5 Å and 3.6 Å, respectively, suggesting that the complex is intrinsically flexible with unrestrained symmetry (Figs. 1D, S2 and Table S1). The backbone of the polypeptide, as well as most side chains, were clearly defined (Fig. 1D), allowing an atomic model of the terminase complex to be built *de novo* (Fig. S3).

Herpesvirus terminases are known to be a member of the additional strand, conserved glutamate (ASCE) superfamily of ATPases (Berger, 2008) however, our cryoEM structure of the herpesvirus hexameric terminase assembly shows that its size, with an external diameter of ~225 Å and a height of ~100 Å (Fig. 1E), is substantially larger than the dimensions of most ASCE members (Miller and Enemark, 2016). Interestingly, the dodecameric assembly is a pair of stacked hexamers, suggesting a high inclination for terminase complex to assemble into a hexameric ring *in vitro* (Fig. S3). Despite of associations with a dodecameric portal (White et al., 2003), the terminase assembly is expected to be a hexmeric helicase type structure due to the matched channel diameter with the portal channel (See below). The central channel of the hexameric ring, having a funnel-like shape with an internal diameter of 39 Å, can possibly bind and translocate the dsDNA substrate using specialized basic residues that project into the central channel (Fig. 1E). Expectedly, the central channel of the hexameric ring has a similar internal diameter to that of the dodecameric portal in herpesviruses (Nan Wang et al., 2020), also suggesting the role in dsDNA translocation. Each subunit of the hexameric ring is a heterotrimer formed by three proteins (pUL15, pUL28 and pUL33) interdigitating with each other (Fig. 1E).

### pUL15 organization and structure

**Figure 2:**
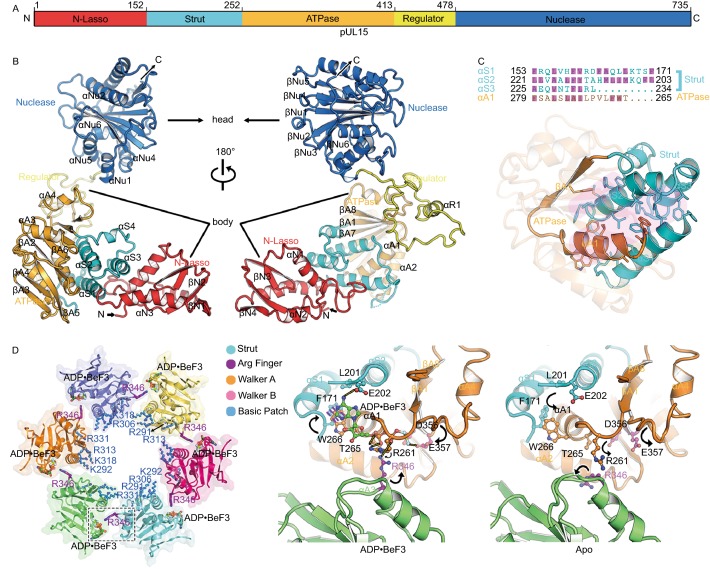
**pUL15 organization and structural features of the ATPase ring**. (A) Schematic diagram of domain organization of pUL15. (B) Two different views of overall structure of pUL15. Domains, N terminus, C terminus and secondary structural elements of pUL15 are labeled. Color scheme is the same as Fig. 2A. (C) Fixation of the ATPase by the strut through hydrophobic interactions. Side chains are shown for hydrophobic residues in the helix bundle (bottom), with their identities marked (magenta) in the sequence (top). (D) Structural features of the ATPase ring (Left) and enlarged view of the active site (Right). Each subunit of the ATPase ring is depicted in a different color, basic residues lining the central channel are represented as blue sticks, ADP•BeF3 and putative arginine finger are shown as sticks. Conformational changes upon ATP hydrolysis and release are marked by arrows, the F171 benzene ring rearranges to stack with W266 indole and the adenine base (blue dashes). Hydrogen bonds are shown as yellow dashes

pUL15 (735 residues in length), the most conserved gene within the family *Herpesviridae* and ~50% larger than its homolog TerL from bacteriophages, is at least a bi-functional ATPase/nuclease that has been demonstrated to be critical for both the cleavage and packaging of viral DNA (Heming et al., 2017). pUL15 folds into an “L” shaped structure, containing five functional domains: N-lasso (residues 1 to 152), Strut (residues 153 to 252), ATPase (residues 253 to 413), regulator (residues 414 to 478) and Nuclease (residues 479 to 735), that constitute the top (nuclease domain) and body regions (Figs. 2A, 2B and S4). The N-lasso domain includes the N-terminal 152 residues, in which a four-stranded β-sheet and three helices are connected by extended loops to form a closed circle, lassoing the pUL28 (See below) (Figs. 2B and S5). The strut domain consists of three α-helices and one short 3_10_ helix, which appears to fix the ATPase domain “backbone” by the formation of an inter-domain four-helix bundle via extensive hydrophobic interactions (Fig. 2C). The regulator, a 65-residue domain, comprising swirling loops and one short helix connect the ATPase and nuclease domains (See below).

### pUL15 ATPase domains form a channel conducive to DNA translocation

DNA translocation is ATP-dependent and the pUL15 ATPase domain converts chemical energy obtained from the hydrolysis of the γ-phosphate bond of ATP into a mechanical force and physical motion, a process usually involving conformational changes of the motor building block. However, many bacteriophage TerLs display incomplete ATP-hydrolysis activities *in vitro* (Sun et al., 2007; Zhao et al., 2013a; Hilbert et al., 2015), presumably because either they do not assemble into a ring-like structure, or they are not activated by proper substrates. In our structures, six copies of the ATPase domain are arrayed around the interior of the ring, forming a “funnel” like structure with a central channel (Fig. 2D). Remarkably, a large basic patch of six residues, R291, K292, R306, R313, K318 and R331, that are highly conserved in herpesviruses and bacteriophages, is observed lining the central channel, which is believed to bind DNA during translocation (Figs. 2D, S4 and S6). Our structures support previously reported experimental observations that the ATPase domain, rather than the nuclease domain, can tightly grip DNA (Hilbert et al., 2017).

### Identification of the *trans*-arginine finger necessary for DNA translocation

Like other ASCE family members, the ATPase active center comprises the Walker A (or P-loop) and B motifs from one protein subunit (Fig. 2D), while ATP hydrolysis is triggered by the insertion of an ‘‘arginine finger’’ into the active center. Whether the terminase arginine finger is located in the same protein subunit as the Walker A and B motifs or is contributed by a neighboring protein subunit is still debatable (Sun et al., 2008; Zhao et al., 2013a; Hilbert et al., 2015; Xu et al., 2017a). Structural comparisons of our structures of the terminase assemblies in the absence and presence of ADP•BeF3 show obvious conformational changes at the ATPase active center and the basic patch (Figs. 2D, S6 and S7). Upon binding the ATP mimic, the P-loop residues R261 and T265 rotate to contact the γ-phosphate mimic, whereas the adenine base is sandwiched between the aromatic rings of residues W266 and F171 (Fig. 2D). Additionally, residues E202 and E357 alter their configurations to make H bonds with ADP•BeF3. Interestingly, residue R346 from an adjacent protein subunit inserts into the ATP binding pocket and interacts with the γ-phosphate, contributing the third positively charged residue (R261, R262 and R346) at the ATPase active center like other ASCE family members (Hilbert et al., 2015; Miller and Enemark, 2016) (Fig. 2D), which indicates that residue R346 might be a trans-acting arginine finger candidate. As expected, the putative arginine finger R346 is largely conserved in terminases across the herpesvirus and bacteriophage families (Fig. S4). Consistent with our structural analysis, the R346A mutant did not alter its oligomeric assembly, but completely abrogated ATPase activity, demonstrating it as the *bona fide* arginine finger (Fig. 1C). In support of this, the residue corresponding to herpesvirus residue R346, in TerL from phage P74-26, R139, was verified as the arginine finger by a biochemical complementation assay (Hilbert et al., 2015).

### The pUL15 nuclease domains is distal from the DNA translocation channel during translocation mode

After a unit length genome is translocated, the terminase utilizes its nuclease domain to initiate a second site-specific cleavage step, leading to the dissociation of the DNA concatemer from the filled capsid and the start of the DNA packaging into another procapsid (Sun et al., 2010). Recent evidence has demonstrated that the nuclease domain is dispensable for DNA binding, and that the ATPase domain is the primary site for DNA binding and is required for nuclease activity, suggesting a concerted mechanism for DNA cleavage (Hilbert et al., 2017). In our structures, the nuclease domain resembles closely the RNase H-like endonucleases with conserved catalytically active residues like D509, E581, D706 and D707 (Selvarajan Sigamani et al., 2013; Xu et al., 2017b). However, the nuclease domains are distal from the central channel (~45 Å from the exterior of the channel) and the catalytically active residues are oriented towards the sides of the channel in the hexameric ring (Fig. S6), which suggests that head-full sensing relayed to the terminase complex through the portal (Nan Wang et al., 2020) would reorient the nuclease domain to be proximal to the central channel for DNA cleavage. The regulator linking the ATPase and nuclease domains might be involved in this re-arrangement of the nuclease domain (Fig. S6).

### pUL28 structure and its interactions with pUL15 and pUL33

**Figure 3:**
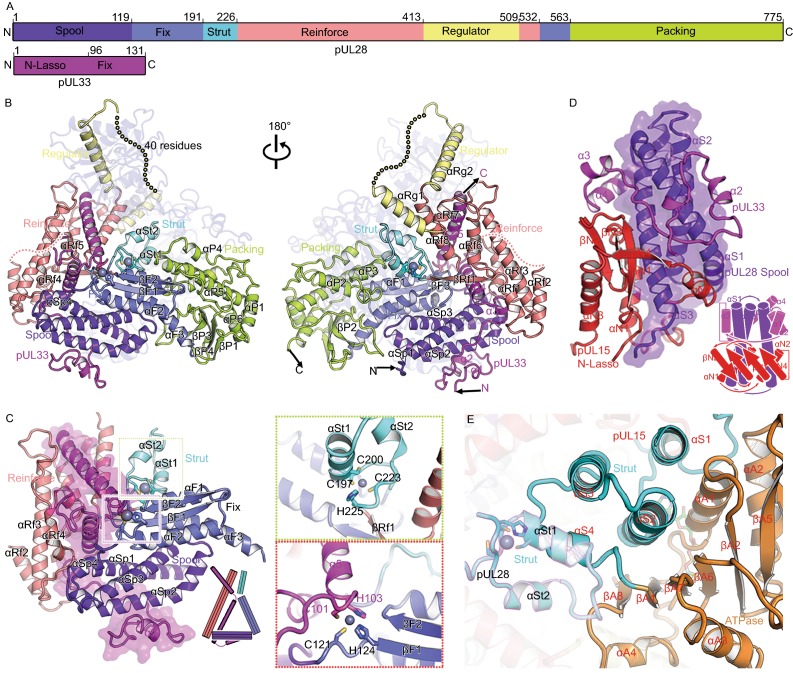

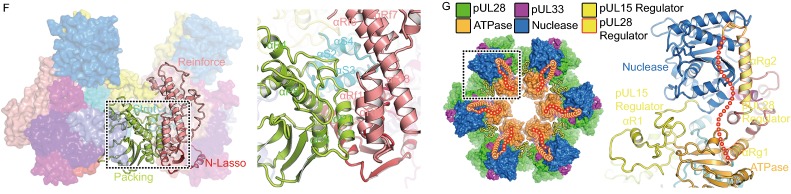
**Structural basis for assembly of the terminase complex**. (A) Schematic diagram of domain organization of pUL28 and pUL33. (B) Views of overall structure of the terminase complex. Domains, N terminus, C terminus and secondary structural elements of pUL28 and pUL33 are labeled. Color schemes for pUL28 and pUL33 are the same as Fig. 3A. pUL15 is colored in blue with 70% transparency. Residues 433–477 that are disordered in the structure are labeled as dots. (C) The “thread a needle” interaction mode between pUL33 and pUL28. A model shown at the right-bottom depicts the interaction mode. Insets illustrate two zinc-finger structures. (D) The “spool of wires” assembly mode of three N-terminal domains of the terminase complex. A model shown at the right-bottom depicts the assembly mode. (E) Double fixation of the ATPase by two sets of three-helix bundle from two strut domains. pUL28 and pUL15 are highlighted in magenta and black outlines. Secondary structural elements from pUL15 are labeled in red. (F) The interactions between two adjacent subunits of the hexameric terminase assembly. The packing domain shows tight contacts with the N-lasso, reinforce and strut domains from the neighboring subunit in the black inset. Color scheme is same as in Figs. 2A and 3A. (G) The proposed regulatory re-arrangement of the nuclease using two sets of “regulators” from pUL15 and pUL28. Left: the location of two “regulators” in the hexameric terminase assembly; Right: zoom-in view of two “regulators” within one terminase complex. The regulators from pUL28 and pUL15 are highlighted in magenta and black outlines

Unlike bacteriophages, herpesviruses use three proteins (pUL15, pUL28 and pUL33) to assemble the terminase complex and mutated viruses lacking a functional version of any of these three can neither initiate DNA packaging nor cleave concatemeric DNA (Reynolds et al., 2000; Yang et al., 2008). The 85-kDa pUL28 (which has no sequence or predicted structural similarities with bacteriophage TerS) and 15-kDa pUL33 remain enigmatic for their structures and functions. pUL28 folds into six distinct domains: spool (residues 1–119), fix (residues 120–191 and 533–563), strut (residues 192–226), reinforce (residues 227–413 and 510–532), regulator (residues 414–509) and packing (residues 564–775) (Fig. S8) based on its roles in initializing the assembly of the terminase complex, and all domains are engaged in extensive associations with pUL15 (interaction area ~6,700 Å^2^) (Fig. 3A and 3B). Although pUL28 (or its homolog pUL56 in human cytomegalovirus, HCMV) was reported to recognize viral *pac* DNA sequence, and have both ATPase and nuclease activities (Bogner et al., 1998; Adelman et al., 2001; Hwang and Bogner, 2002; Scholz et al., 2003), structural features typical for ATPase and nuclease activities are not found in our pUL28 structures. However, pUL28 and its homolog pUL56 are the targets of various Food and Drug Administration approved anti-viral drugs by blocking the formation of infectious virions (Bogner, 2002; Melendez and Razonable, 2015), highlighting a role for pUL28 in ensuring correct folding or assembly of the complex.

pUL33, consisting primarily of five helices (Figs. 3B and S8), threads through a compact “triangle” formed by the spool, fix, strut and reinforce domains of pUL28, thus firmLy stabilized by extensive interactions with pUL28 (contact area ~4,500 Å^2^) (Fig. 3C). In turn, the pUL28 N-terminal spool domain is twined around by two N-lasso domains of pUL15 and pUL33, further facilitating the assembly of the complex (Fig. 3D). Unexpectedly, our structure reveals an intermolecular Zn finger formed by residues C121 and H124 from pUL28 fix domain, and residues C101 and H103 from pUL33, which are highly conserved across the herpesvirus family. Furthermore, the pUL28 fix domain, comprising a three-helix-bundle plus an antiparallel two-stranded β-sheet, appears to fix the orientation of the pUL33 C-domain (Figs. 3C and S9). The pUL28 reinforce domain, composed of several helices, contributes to the third side of the triangle, thus further strengthening the interactions between pUL28 and pUL33 (Figs. 3C and S9). Two clusters of three-helix bundles formed by the strut domains of pUL15 and pUL28 support each other, along with a second intramolecular Zn finger (residues C197, C200, C223 and H225 from pUL28), immobilizing the “backbone” of the ATPase domain (Fig. 3E). The pUL28 C-terminal packing domain makes extensive contacts between adjacent subunits through interactions with the N-lasso, strut of pUL15 and pUL28 reinforce domain (Fig. 3F). Acting as core organizers, pUL28 initializes and ensures correct assembly of the complex.

### Rearrangement of the nuclease domains mediated by the regulator leading to DNA cleavage

As with that of pUL15, the regulator domain of pUL28 communicates with both the ATPase and nuclease domains via two helices that are linked by a long flexible (disordered/low density) loop (Fig. 3G). Notably, the two regulator domains, one from pUL15 and the other from pUL28, are positioned on the two sides of the ATPase and nuclease domains, as if to act as hinges to regulate conformational changes of the nuclease (Fig. 3G). We suggest that, upon receiving the “head-full” signal either from the portal (Nan Wang et al., 2020) or ATPase domain (possibly recognizing specific DNA sequences), the nuclease undergoes conformational changes around these two distinct hinges to switch modes from translocation to cleavage. Further, superimpositions of our pUL15 and four full-length structures of TerLs in bacteriophages show various domain arrangements for the nuclease, which are mediated by the linker (We have called it regulator) (Fig. S10). Models for the above four TerL rings generated based on our hexameric ring also show two distinctive configurations, with the nuclease domain in one far from, and in the other proximal to, the central channel, corresponding to the translocation and the cleavage mode, respectively (Fig. S10).

## Discussion

**Figure 4:**
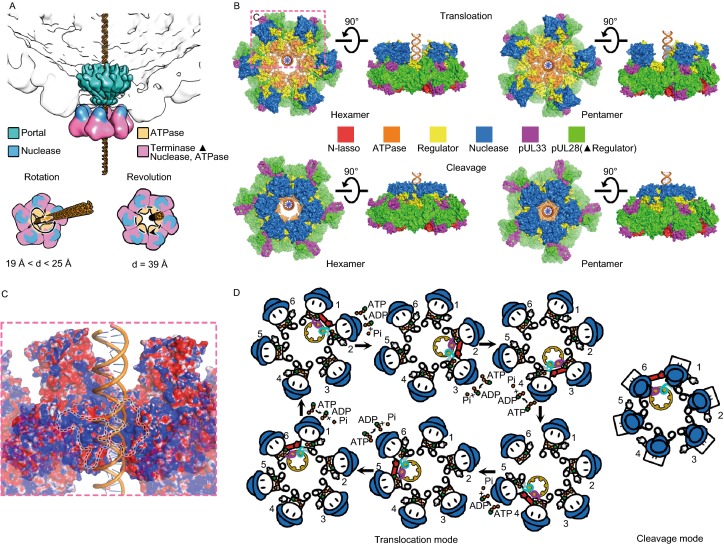
**Proposed model of DNA translocation and cleavage.** (A) Structure-based analysis of rotation or revolution motions during DNA translocation. The dodecameric portal, nuclease and ATPase domains are colored in green, blue and orange respectively. The diameters of ATPase channels of hexameric and modeled pentameric terminase rings are shown. (B) Proposed models for DNA translocation and cleavage. The revolution (left) and rotation (right) mechanisms for DNA translocation are proposed based on assemblies of hexameric and pentameric rings. Upon completion of DNA translocation, the nuclease rearranges itself to enter “cleavage mode”. Notably, in hexameric ring (left), the nucleases (as well as the catalytically active residues) are in proximity to DNA, while the nucleases seem unlikely to cleave DNA due to spatial disconnection in pentameric ring (right). (C) Two basic patches between adjacent terminase complexes. 1 and 2 patches represent the purple and cyan ones in Fig. 4D. (D) Illustration of the motion of DNA revolving inside the ATPase channel and DNA cleavage upon completion of packaging. The two feet (purple and cyan) represent two basic patches from the ATPase, the hand indicates the arginine finger (the red one represents the activated arginine finger) and the hat is the nuclease. Upon completion of DNA packaging the nuclease domains (blue hats) rearrange, entering the “cleavage mode”

The past 30 years have witnessed fervent debates over whether the viral genome packaging motor is a hexamer or a pentamer (Guo et al., 1998; Zhang et al., 1998; Sun et al., 2007; Zhao et al., 2013b; Hilbert et al., 2015, 2017). A tug of war also remains concerning the mechanism of packaging, between a model involving rotation of components ‘screwing’ the DNA into the head, and a model where the DNA revolves around a larger cavity during translocation (Sun et al., 2007; Guo et al., 2013; Zhao et al., 2013b). Our hexameric structures of the herpesvirus terminase complex with the central channel bigger (39 Å in diameter) than the diameter of the B-form dsDNA (~20 Å in diameter) favors the revolution model, which is consistent with the internal diameter of the herpesvirus portal (~36 Å in diameter) (Nan Wang et al., 2020) (Fig. 4A and 4B). In the rotational model, at least one of the three components—portal, terminase ring and dsDNA—must rotate during dsDNA translocation. Hypothetically, a pentameric ring structure could be created by rearranging the subunit resolved in our hexameric structure with spatial restrains including maintenance of reasonable contacts without severe clashes between adjacent subunits. The diameters ranging from 19 to 24 Å along the path of the central channel of this hypothetical pentameric ring could in theory accommodate the rotation model of DNA translocation (Fig. 4A and 4B). However, due to possible supercoiling, tangling and torque associated with dsDNA compaction, dsDNA translocation through the terminase complex requires sequential signaling and coordinated action from one motor subunit to its neighbor, which is incompatible with the nut-and-bolt type of contacts between the channel and dsDNA in the pentameric ring. This, together with the fact that neither the portal nor terminase assembly rotates during DNA translocation (Hugel et al., 2007), argue strongly against the rotation model of DNA translocation. Our structures support instead the revolution model whereby the hexameric terminase assembly follows a sequential coordination, one-way revolution mechanism to drive dsDNA translocation. During genome translocation, dsDNA could bind to two basic patches (purple for weak binding and cyan for tight binding) from two neighboring subunits’ ATPases (denoted as S1 and S2) with all six (S1-S6) bearing ATP molecules (Fig. 4C). Binding of dsDNA to the S1 purple patch would trigger the rotation of the arginine finger, which constitutes a complete “active site” in S2 and facilitates ATP hydrolysis *in trans* (Fig. 4D). When S2 hydrolyzes ATP, it would undergo a conformational rearrangement, such that the ATPase, in particular the cyan patch, would pivot 15° around the strut, driving the DNA in an upward spiral (Figs. 4D and S6). The conformational change from one ATP hydrolysis event in S2 would propagate to S3, sterically exerting force on S3 such that the two ATPases move in concert, leading to a second upward spiraling of the DNA and “passing” it to the next two basic patches (Fig. 4D). During this process, S1 would withdraw its arginine finger from DNA, completion of ATP-hydrolysis in S2 would detach S2 from DNA and initiate ATP hydrolysis in S3, starting the next cycle for DNA translocation (Fig. 4D). During the translocation mode, the nuclease active site is sequestered from DNA by interactions of pUL15 with the portal, preventing pre-mature cleavage (Fig. 4D). Immediately prior to completion of packaging, head-full signal sensed by the capsid proteins and relayed through the portal (Nan Wang et al.,^2020^) triggers pUL15 to undergo a regulator mediated domain rearrangement of the nuclease, converting the terminase complex to the cleavage mode (Fig. 4D).

Since the dawn of humanity, herpesvirus infections have been wide-spread and persistent among human populations, causing inconvenient symptoms and sometimes medically significant complications, such as genital herpes, shingles and cancers. Genome packaging by the terminase complex is essential to, or the Achilles heel of, all herpesviruses. Indeed, several FDA-approved anti-herpesvirus drugs target the terminase complex. The first atomic structure of a herpesvirus terminase complex presented here now provides a blueprint for future mode-of-action studies for these drugs and shall help development effort for better terminase inhibitors against herpesvirus infections.

## Methods

### Cloning, expression and purification of the terminase complex

Gene HSV1-pUL15/mutant (R346A) (residues 1–737) was cloned in the pFastBac-HTA vector which bears an N-terminal 6xHis tag. Genes HSV1-pUL28 (residues 1–787) and HSV1-pUL33 (residues 1–132) were cloned in the pFastBac-Dual vector under the polyhedrin and p10 promoters, respectively. All the three proteins were expressed simultaneously in Spodoptera frugiperda 9 (Sf9) cells by co-infecting the above two recombinant baculoviruses. The culture was processed after 3 days of incubation. The cells were centrifuged for 20 min at 4 °C (3,000 rpm). The pellet was resuspended in 50 mL lysis buffer (20 mmol/L Hepes, 300 mmol/L NaCl, pH 7.5) and cells homogenized by freezing and thawing three times. The solution was centrifuged for 2 h at 4 °C (34,000 rpm), and the pellet discarded. The supernatant was loaded onto a 15 mL gravity column (Bio-Rad) containing 5 mL of pre-equilibrated Ni-NTA Agarose (Qiagen) beads. Then the beads were washed with the washing buffer containing 20 mmol/L Hepes, 500 mmol/L NaCl, pH 7.5 for 20~30 column volumes and further washed by using washing buffer containing 20 mmol/L imidazole until no traces of the protein were detected in the flow-through. Proteins bound to the column were eventually eluted in a buffer containing 20 mmol/L Hepes, 300 mmol/L NaCl and 200 mmol/L imidazole, pH 7.5, then concentrated (Amicon Ultra-15 30,000 MWCO, Millipore) to approximate 500 μL and loaded onto a Superose 6 gel filtration column (GE Healthcare) equilibrated with lysis buffer. Fractions for each peak were collected separately, concentrated to 1 mg/mL for cryoEM sample preparation.

### Sedimentation velocity experiments

Sedimentation velocity assays were performed on a Beckman XL-I analytical ultracentrifuge at 20 °C, which has been described previously (Wang et al., 2012; Wang et al., 2015; Zhu et al., 2018b). The protein sample was diluted with lysis buffer (20 mmol/L Hepes, 300 mmol/L NaCl, pH 7.5) to 400 μL at A280 nm absorption of ~0.8. Samples and reference solvents were loaded into double-sector quartz cells, and mounted in a Beckman four-hole An-60 Ti rotor. Data were collected at 20,000 rpm 6h at a wavelength of 280 nm and analyzed using the SEDFIT software.

### ATPase assays

ATPase assays were performed at room temperature with buffer solutions containing 2 mmol/L ATP, 50 mmol/L Tris-HCl (pH 7.5), 1 mmol/L MgCl_2_, 0.1 mmol/L sodium azide, 150mmol/L sodium chloride, 0.1 μmol/L wild-type or mutant (R346A) hexameric terminase assembly or 0.1 μmol/L monomeric terminase complex. Measurement of ATP hydrolysis was based on a spectrophotometric shift in the maximum absorbance of the substrate from 330 nm to 360 nm, resulting from the enzymatic conversion of 2-amino-6-mercapto-7-methylpurine riboside (MESG) by purine nucleoside phosphorylase (PNP) in the presence of Pi (EnzChek Phosphate Assay Kit). The measurements were performed in a Microplate Reader (VARIOSKAN FLASH, Thermo Scientific), and the ATPase activities were calculated at the early time points when the yield of product increased linearly.

### CryoEM sample preparation and data collection

3.5 μL aliquots of purified terminase complex (1 mg/mL) or terminase complex plus ADP•BeF3 and *pac*2 DNA (58 bp) were applied to previously plasma-cleaned UltrAuFoil 0.6/1.0 300 mesh grids and blotted once for 5 s after a wait time of 15 s. CryoEM data sets were collected at 300 kV with a Titan Krios microscope (FEI). Movies (30 frames, each 0.2 s, total dose 60 e^−^Å^−2^) were recorded using a K2 detector with a underfocus range of 1.5 to 2.5 μm. Automated single-particle data acquisition was performed with SerialEM (Mastronarde, 2005), with a nominal magnification of 22,500× in super-resolution mode, which yields a calibrated pixel of 1.04 Å.

### Image processing

Frames in each movie were motion-corrected and combined to a micrograph, leading to 1,717 for the apo terminase assembly and 702 for ADP•BeF3-bound termianse assembly used for image processing. Out of these, 1,692 micrographs for the apo terminase assembly and 702 for the ADP•BeF3-bound terminase assembly showing visible CTF rings beyond 1/5 Å in their spectra were selected for further processing. The defocus value for each micrograph was determined using Gctf (Zhang, 2016). Particles were boxed using Gautomatch. Multiple rounds of 2D classification were performed to discard bad particles. Comparisons of 2D classification results from the terminase assemblies in absence and presence of ADP•BeF3 and *pac*2 DNA show no notable structural differences, suggesting densities for *pac*2 DNA are invisible. The initial model was generated by cisTEM (Grant et al., 2018). Two rounds of 3D classification with C1 and C6 symmetry separately were performed to further select the particles for final refinement, in which maps for hexameric and dodecameric assemblies were obtained. In line with the results of 2D classification, densities for *pac*2 DNA are invisible in the map of the terminase assemblies in presence of ADP•BeF3 and *pac*2 DNA, which was reconstructed with C1 symmetry. The resolutions of the final maps for the apo-hexamer, apo-dodecamer, ADP•BeF3-hexamer and ADP•BeF3-dodecamer by imposing C6 symmetry are 3.9 Å (43,188 particles), 4.6 Å (10,631 particles), 4.4 Å (9,982 particles) and 4.6 Å (3,890 particles), respectively using the “gold” standard Fourier shell correlation (FSC) = 0.143 criterion. The dodecameric assembly consists of two layers of the hexameric form.

To further improve resolution we used the block-based reconstruction (Wang et al., 2018; Yuan et al., 2018; Zhu et al., 2018a; Wang et al., 2019). The orientation parameters of each particle image (hexamer and dodecamer) determined in Relion were used to guide extraction of the block region (~50% bigger than monomer) and the block was refined and reconstructed (Scheres, 2012). After refinement and reconstruction of the block in Relion (Scheres, 2012), the resolution of maps for the apo terminase complex and ADP•BeF3-bound terminase were further improved to 3.5 Å (386,700 particles) and 3.6 Å (42,053 particles), respectively. We noticed that the resolution of the map for the head region corresponding to the nuclease domain in the apo terminase complex was still poor. To solve this problem, a local reconstruction focusing on the head region was carried out, finally yielding a resolution of 4.3 Å for the nuclease. However, the head region is very rigid in the case of the ADP•BeF3-bound terminase.

### Model building and refinement

The ab-initial atomic models of the apo and ADP•BeF3-bound terminase complexes were built *de novo* into density using COOT (Emsley and Cowtan, 2004). Among these, the crystal structure of the pUL15 nuclease (PDB code: 4IOX) (Selvarajan Sigamani et al., 2013) was fitted into the maps of the terminase complex using CHIMERA (Pettersen et al., 2004) and further manually corrected in COOT (Emsley and Cowtan, 2004). Models were further improved by iterative positional and B-factor refinement in real space using Phenix (Afonine et al., 2012) and rebuilding in COOT (Emsley and Cowtan, 2004). Refinement statistics are listed in Table S1.

## Electronic supplementary material

Below is the link to the electronic supplementary material.

Supplementary material 1 (PDF 5596 kb)
