# Supplementary material for: Architecture of the herpesvirus genome-packaging complex and implications for DNA translocation
Source: Protein Cell. 2020 Apr 23;11(5):339–51. doi: 10.1007/s13238-020-00710-0 (PMC7196598; doi:10.1007/s13238-020-00710-0)
Supplement: Supplementary file 1 — Supplementary material 1 (PDF 5596 kb) [file 13238_2020_710_MOESM1_ESM.pdf]

Supplementary Materials for

Architecture of the herpesvirus genome-packaging complex and implications for DNA translocation

Authors: Yunxiang Yang[1,2$], Pan Yang[1$], Nan Wang[1], Zhonghao Chen[1], Dan Su[2], Z. Hong Zhou[3], Zihe Rao[1,4,5*] and Xiangxi Wang[1*]

Correspondence to: X.W. (Email: xiangxi@ibp.ac.cn), or Z.R. (Email: raozh@tsinghua.edu.cn)

This PDF file includes:

Figures S1-S10

Table S1

Supplementary Figures

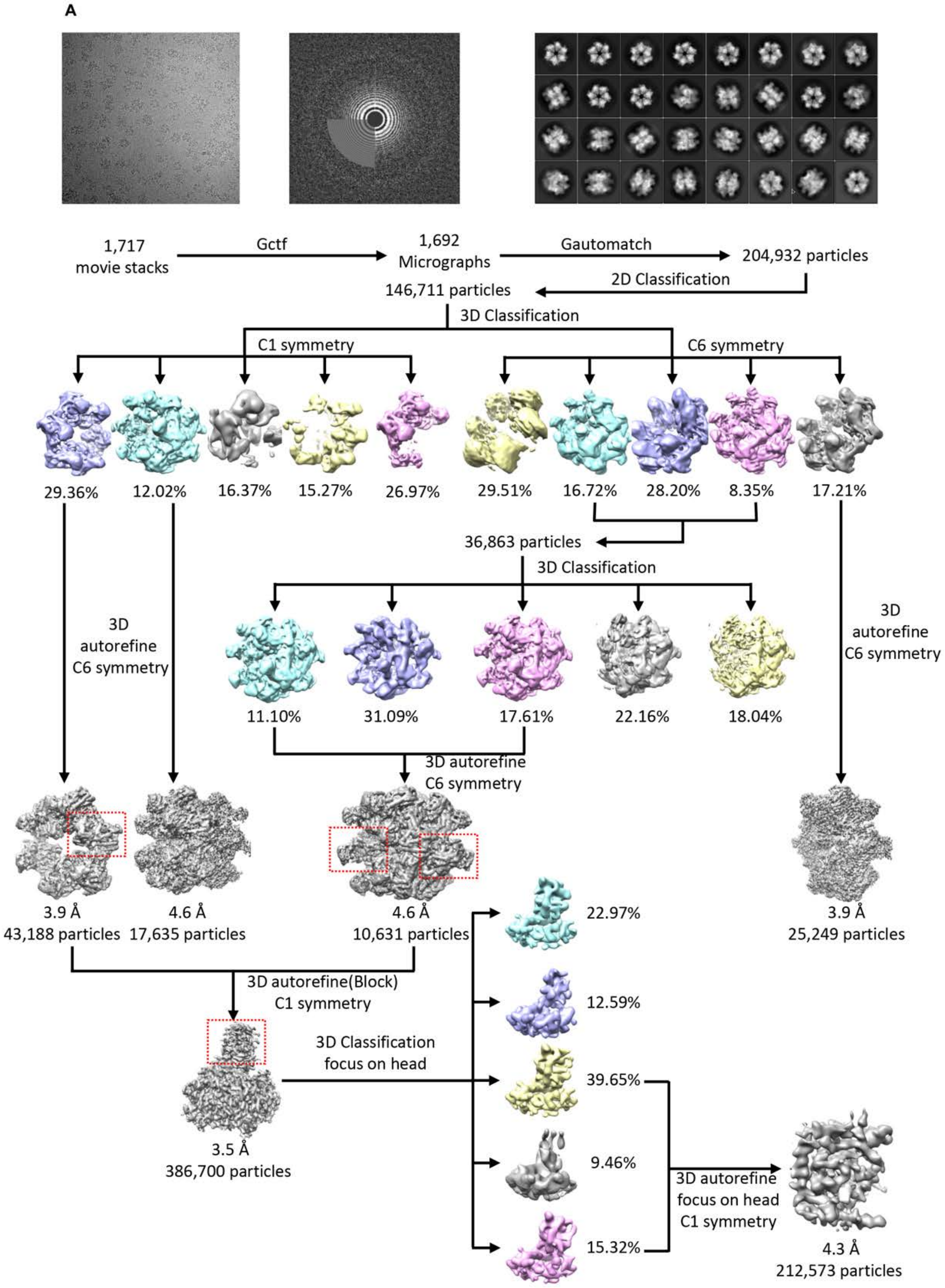

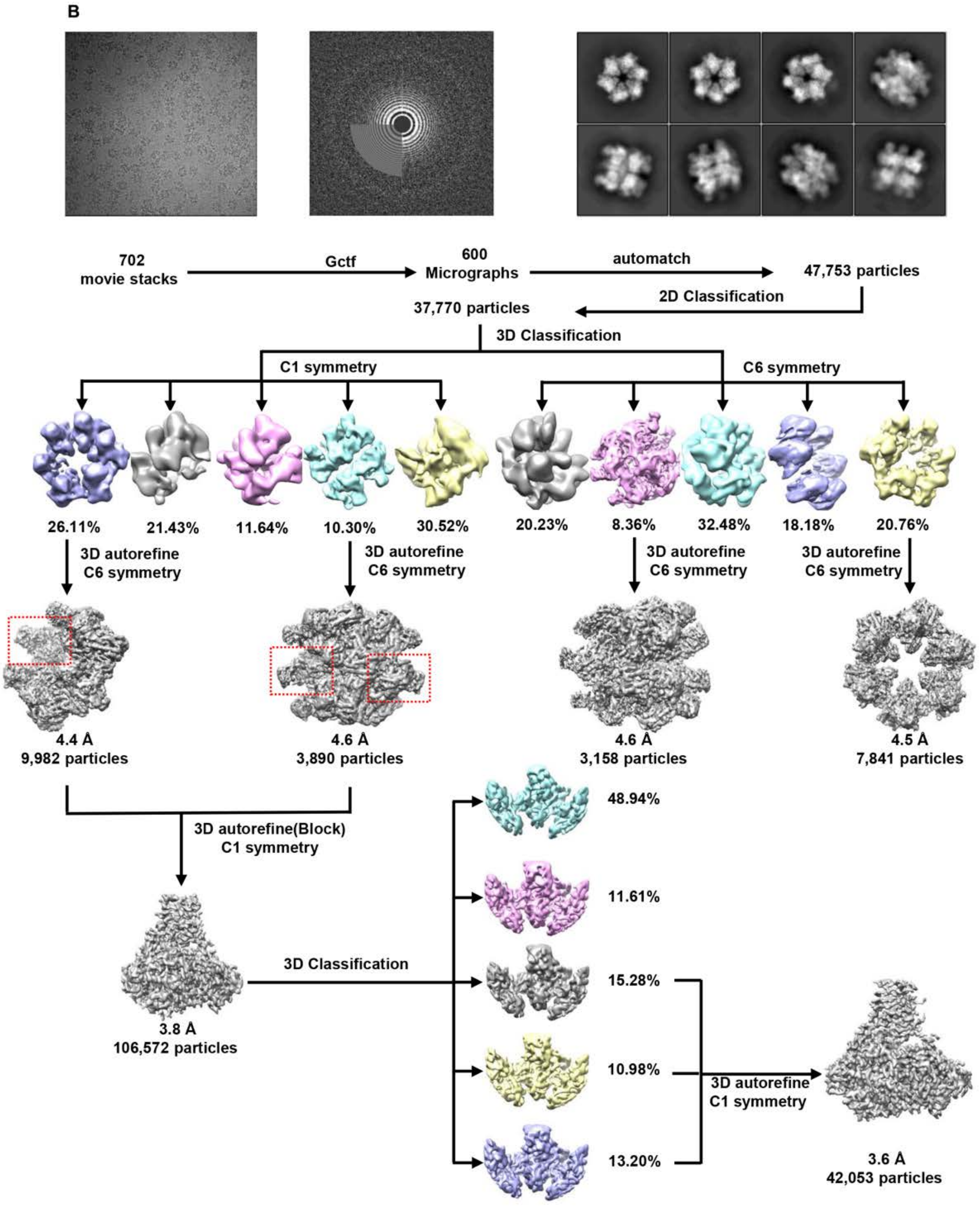

Fig. S1. The flowchart for EM data processing. EM data processing for the apo terminase assembly (A) and the ADP•BeF3-bound terminase assembly (B). CryoEM images of the representative particle (left), the corresponding Fourier transformation (middle), representative 2D classes (right) and the flowchart for EM data processing (Bottom).

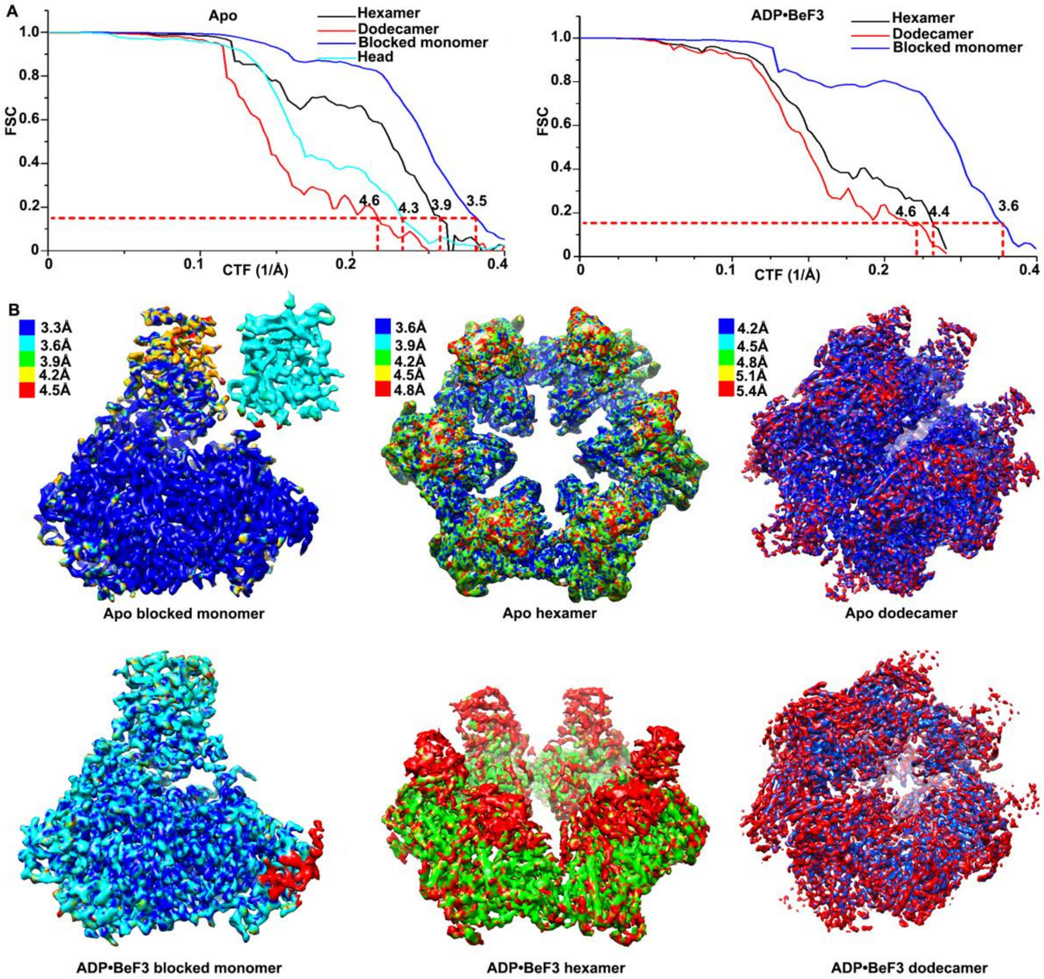

Fig. S2. Resolution evaluation of the cryoEM maps. (A) The gold-standard FSC curves. FSC plots of the apo terminase assembly (left) including the hexamer, dodecamer, blocked monomer and head region, the ADP•BeF3-bound terminase assembly (right) containing the hexamer, dodecamer and blocked monomer based on the FSC=0.143 criterion are shown. (B) The local resolution was evaluated by ResMap *(Kucukelbir et al., 2014).*

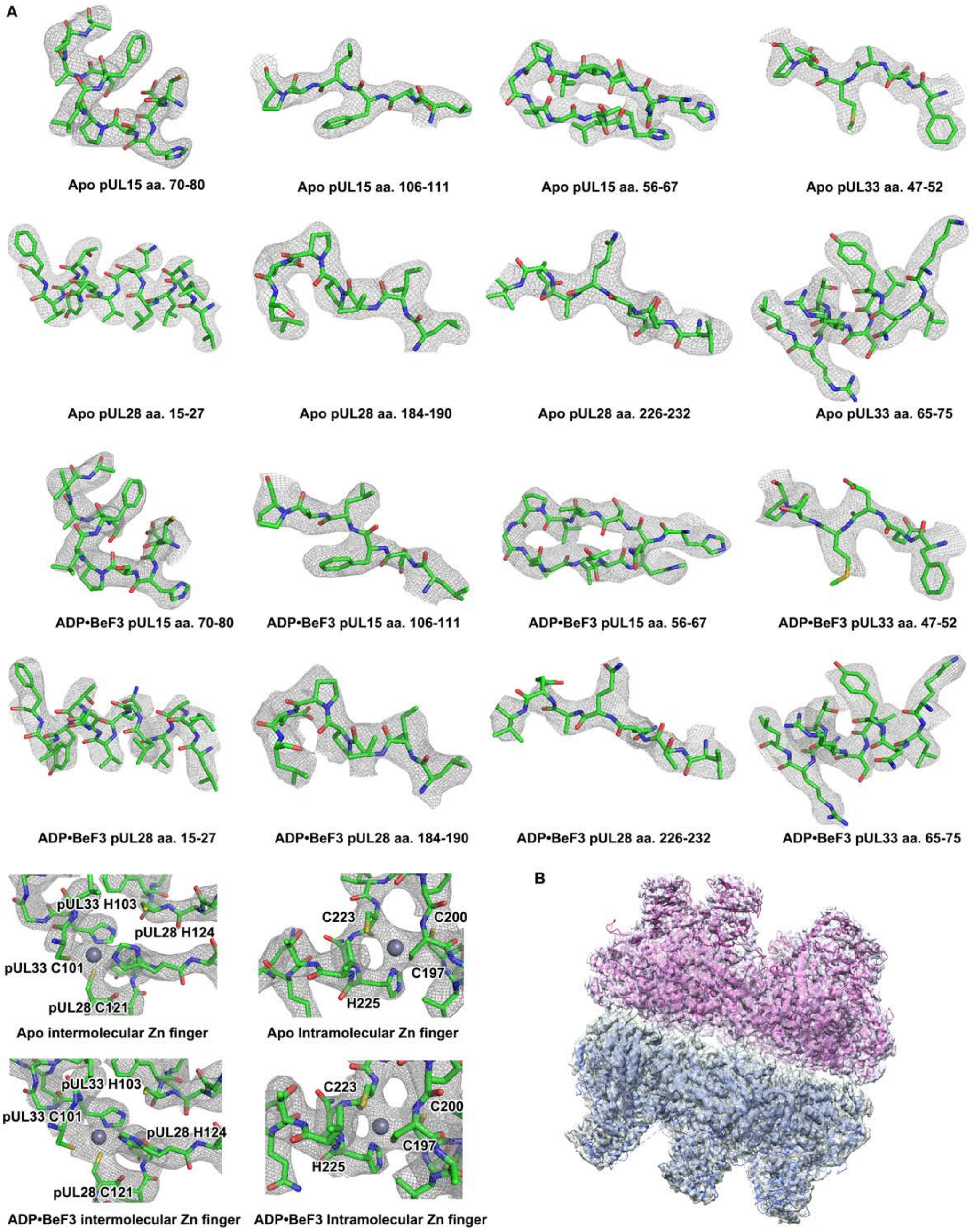

Fig. S3. Density maps and atomic models of the terminase complex. (A) Zoomed-in views of the local densities and illustration of side chain features. Main chain and side chains are colored according to elements (C: green; N: blue; O: red; S: orange). (B) CryoEM map and corresponding model of the dodecameric assembly. The dodecameric terminase assembly is a pair of stacked hexamers in two different colors.

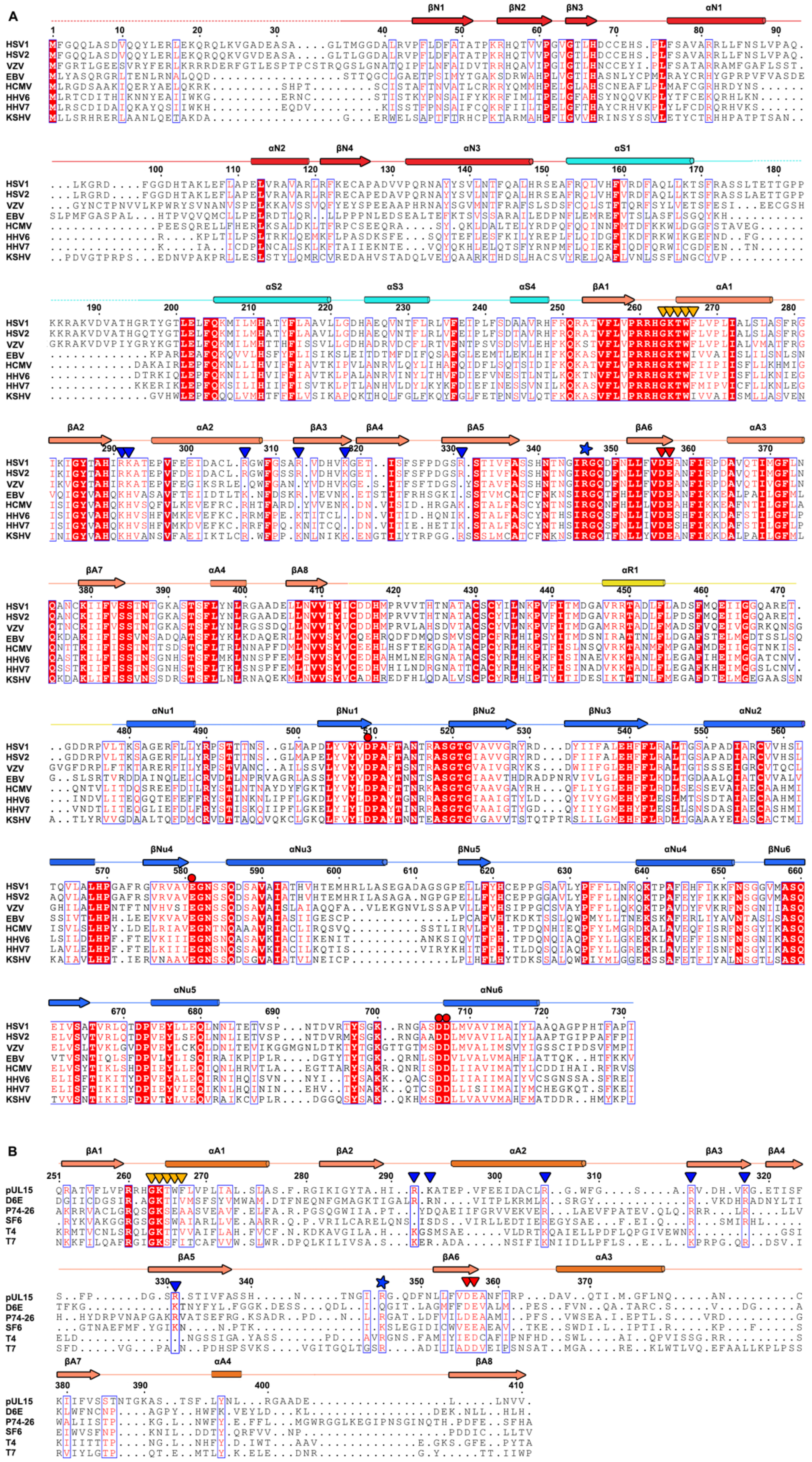
A
HSV1
HSV2
VZV
EBV
HCMV
HHV6
HHV7
KSHV
B
pUL15
D6E
P74-26
SF6
T4
T7

Fig. S4. Multiple sequence alignments of pUL15 and its homologs. Espript representation (Gouet et al., 1999) of sequence alignments of pUL15, its homologs in herpesviruses (A) and its orthologs in bacteriophages (B). Secondary structural assignments on the top are based on the structure determined in this study and colored as in Fig. 2a. The putative arginine finger, basic patch, Walker A, Walker B and active residues of nuclease are marked as a blue star, blue triangles, orange triangles, red triangles and red balls, respectively. Dashed lines represent disordered residues in the structure.

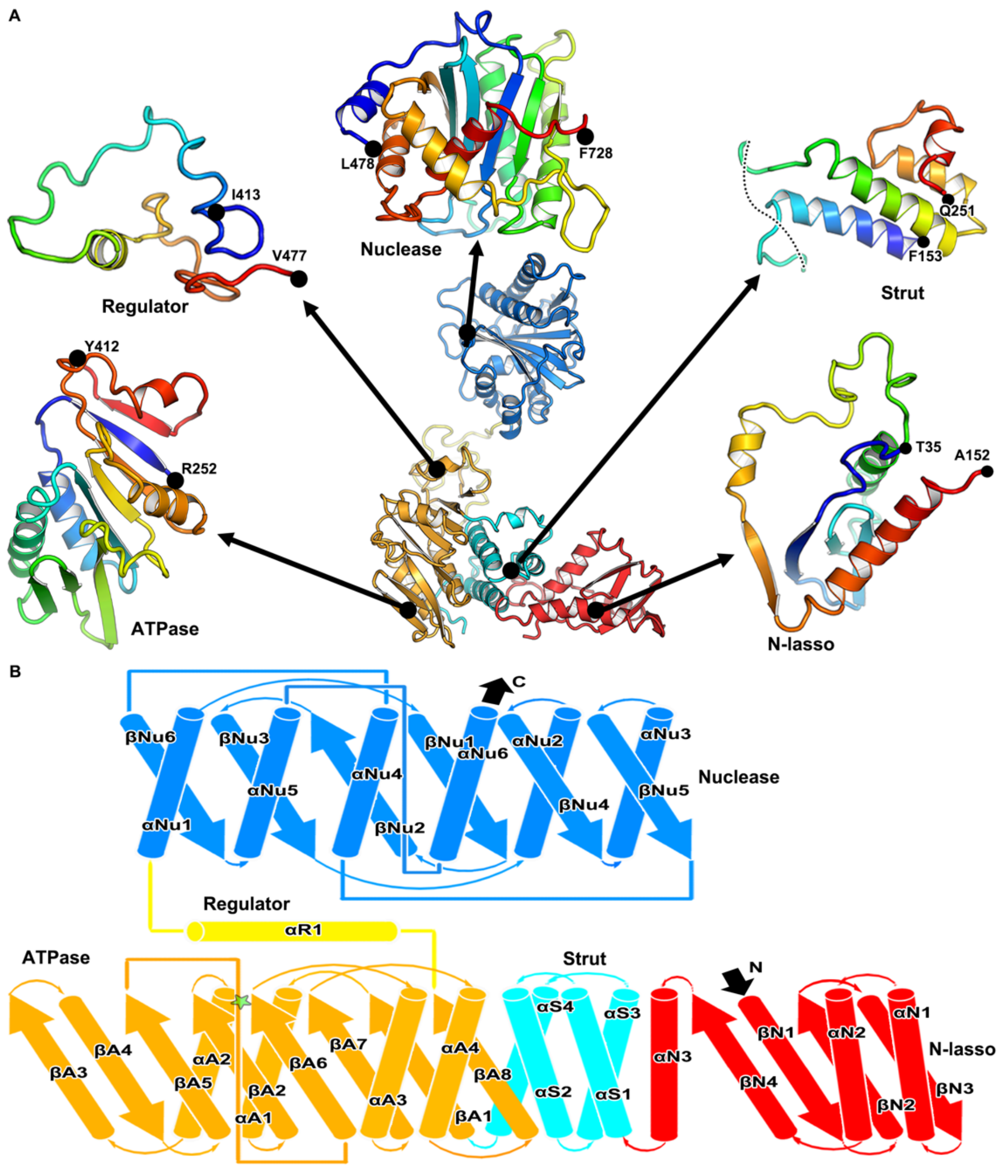

Fig. S5. Overall structure and topology diagram of pUL15. (A) Overall structure of pUL15. The central image is a ribbon diagram colored by domain as in Fig. 2a. Surrounding this are rainbow ribbon representations of individual domains (blue N terminus through green and yellow to red C terminus). (B) Topology diagram illustrating the secondary structural organizations of pUL15. Secondary structural elements, N termini, C termini and ADP•BeF3 binding site are labeled.

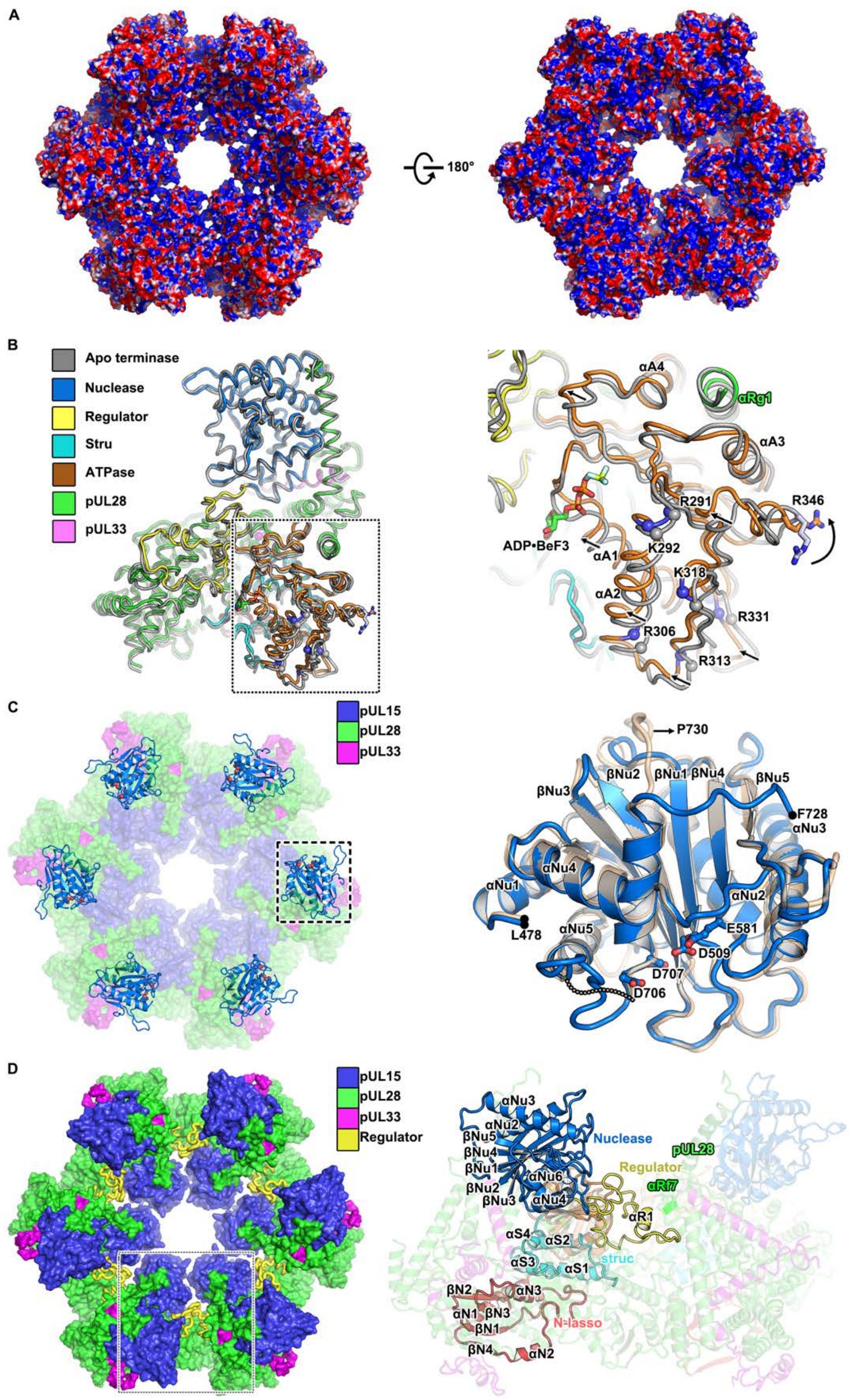

Fig. S6. Characteristics of the hexameric terminase assembly. (A) Top and bottom views of electrostatic surface potential (calculated using APBS in PyMOL) of the hexameric terminase assembly. Red, negative; blue, positive; white, neutral. (B)

Conformational changes upon ATP hydrolysis and release. The terminase complex of the ADP•BeF3-bound structure (colored by individual domains) was superposed onto the apo structure (gray). Enlarged view highlights the major conformational alterations in ATPase. ATP gamma-phosphate mimic and putative arginine finger are shown as sticks, residues in the basic patch are indicated by blue spheres. The black arrows mark the conformational shifts. (C) Domain location of the nucleases in the hexameric terminase assembly. The nuclease is presented as marine cartoon, others are shown as surfaces. Active residues of the nuclease are shown as sticks. Structural comparison of the pUL15 nuclease from the structures determined by cryoEM (marine) and crystallography (wheat, PDB code: 4IOX). The terminal residues of the nuclease are marked. (D) Domain location of the pUL15 regulator in the hexameric terminase assembly. The regulator of pUL15 is presented as yellow cartoon, others are shown as surfaces. Zoomed-in view of two adjacent terminase complexes illustrates the pUL15 regulator is involved in tight contacts between two neighboring subunits via forming two-helix bundle interactions with αRf7 from pUL28. The neighboring subunit is shown with 70% transparency. Color scheme for pUL15 is the same as Fig. 2A, pUL28 and pUL33 are depicted in green and magenta, respectively.

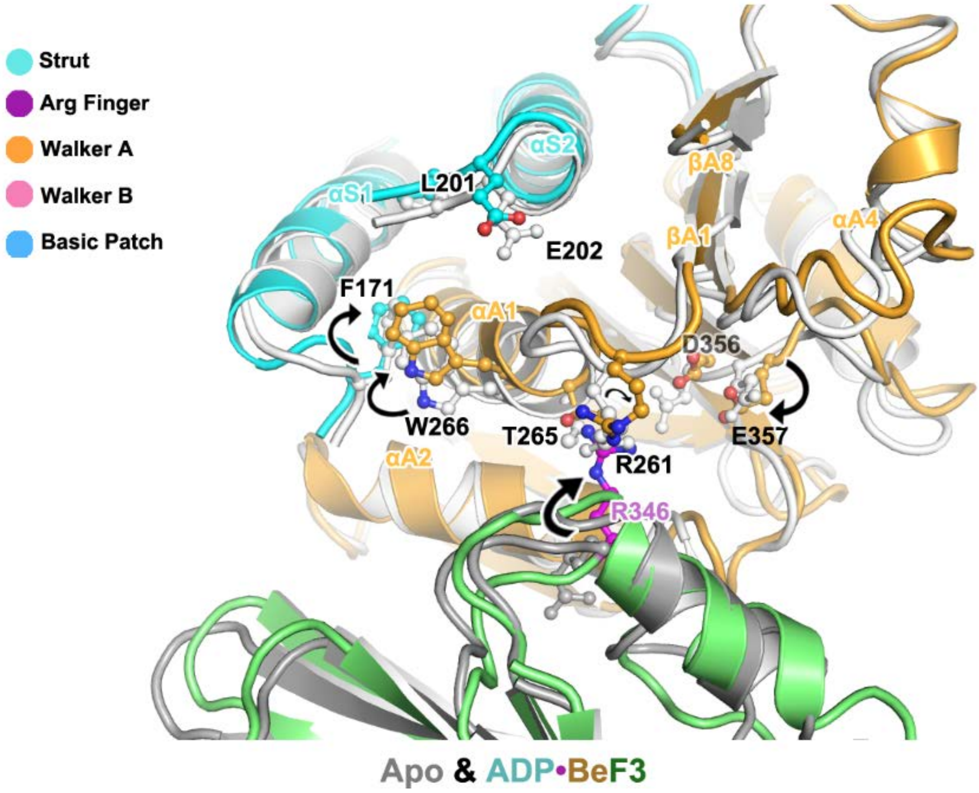

Fig. S7. Structural comparason of the active site in the states of the apo and ADP•BeF3 bound. The active site from Apo state (grey) is superposed to ADP•BeF3 state (colored by domains, color scheme is labeled). Putative arginine finger are shown as sticks and the ADP•BeF3 is not shown. Conformational changes upon ATP hydrolysis and release are marked by arrows.

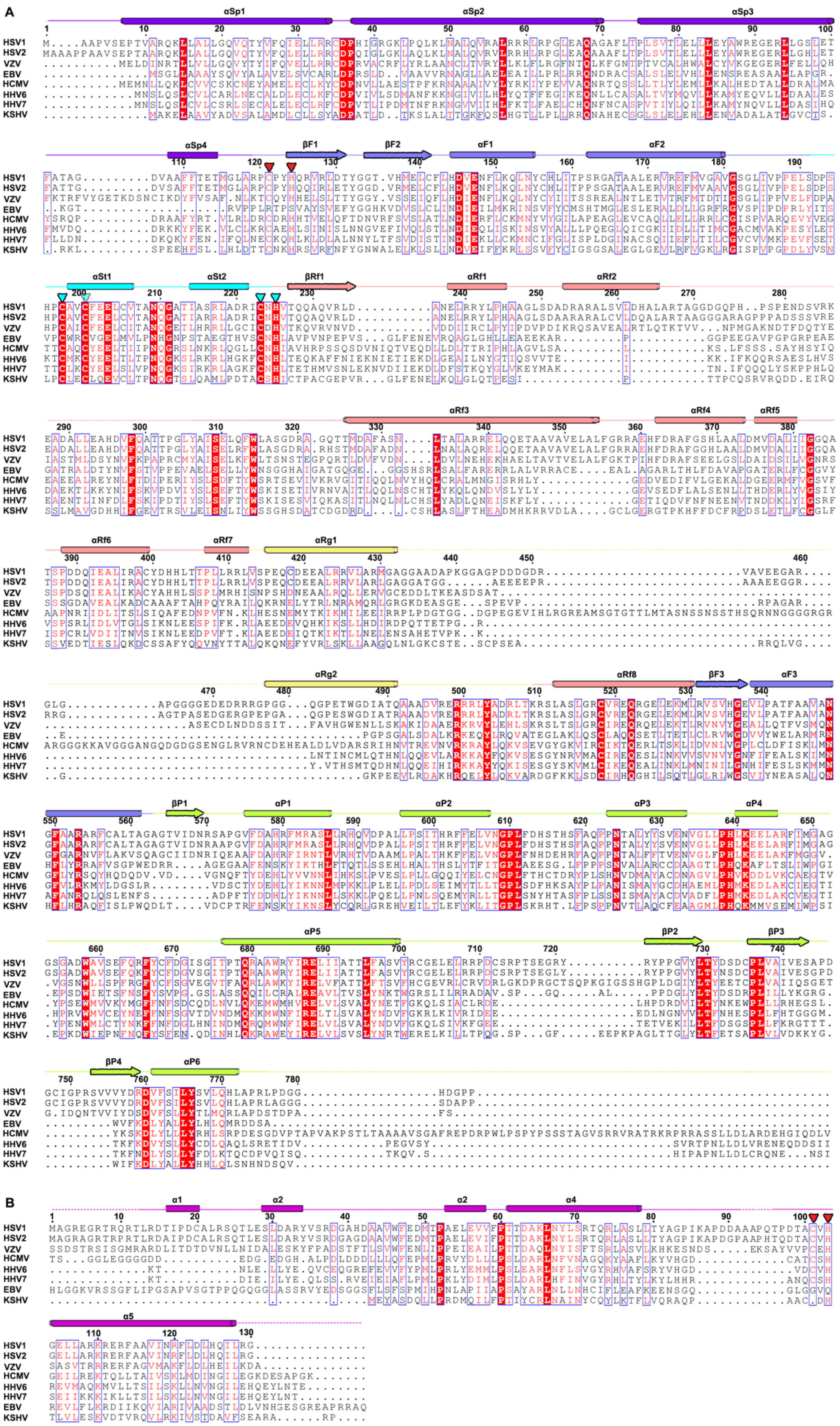
A
B
HSV1
HSV2
VZV
EBV
HCMV
HHV6
HHV7
KSHV
αSp1
αSp2
αSp3
αSp4
βF1
βF2
αF1
αF2
αSt1
αSt2
βRf1
αRf1
αRf2
αRf3
αRf4
αRf5
αRf6
αRf7
αRg1
αRg2
αRf8
βF3
αF3
βP1
αP1
αP2
αP3
αP4
αP5
βP2
βP3
βP4
αP6
α1
α2
α4
α5

Fig. S8. Multiple sequence alignments of pUL28, pUL33 and their homologs. Espript representation of sequence alignments of pUL28 (A) and pUL33 (B) and their homologs in herpesviruses. Secondary structural assignments on the top are based on the structure determined in this study and colored as in Fig. 3a. The residues constituting the Zinc finger within pUL28 and the intermolecular Zinc finger between pUL28 and pUL33 are marked as cyan and red triangles. Dashed lines represent disordered residues in the structure.

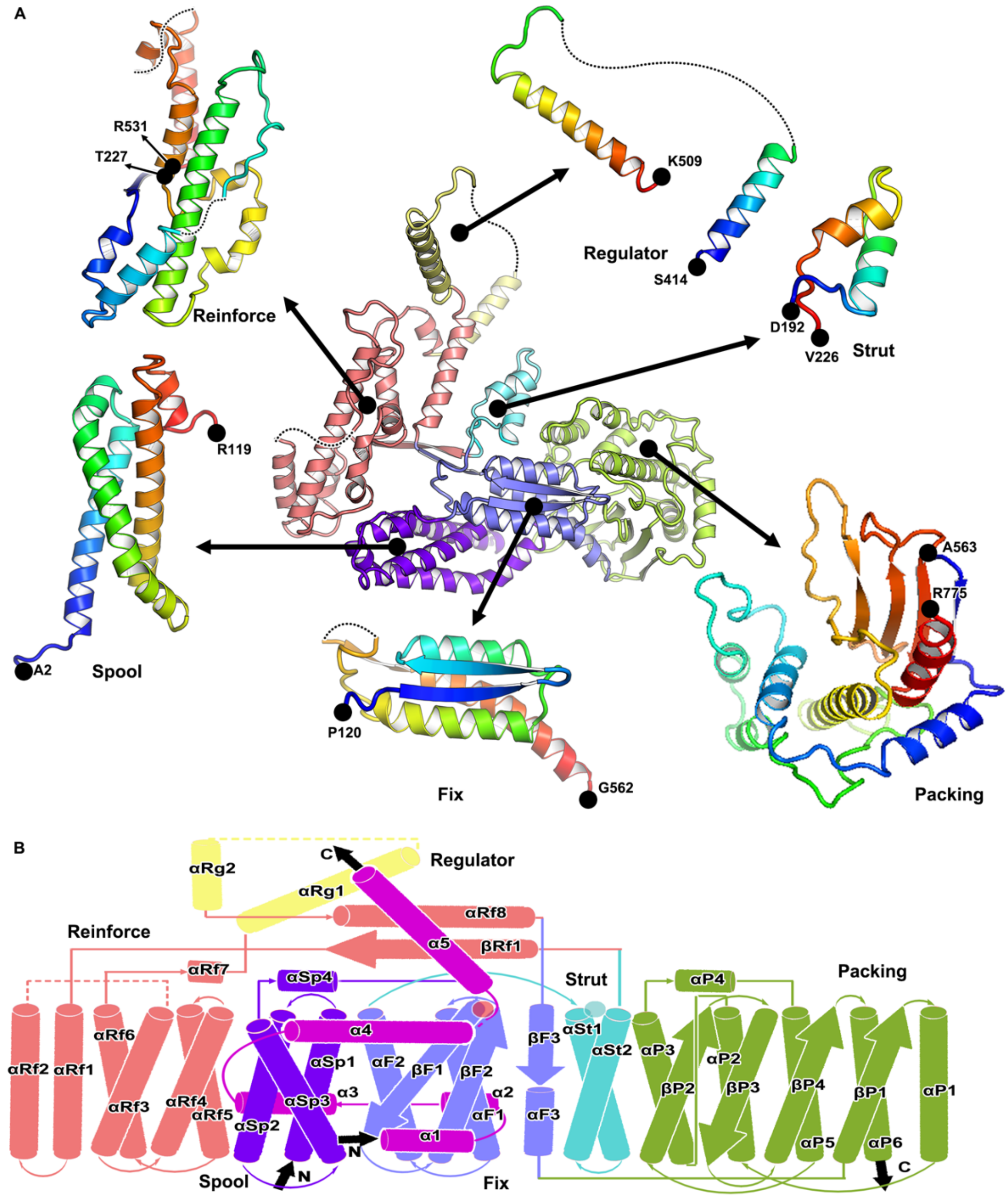

Fig. S9. Overall structure and topology diagram of pUL28. (A) Ribbon diagram of pUL28. The central image shows pUL28 colored as in Fig. 3a. Rainbow ribbon models show individual domains (blue N terminus through green and yellow to red C terminus). (B) Topology diagram illustrating the secondary structural organization of pUL28. Secondary structural elements, N termini, C termini and intramolecular Zinc finger are labeled.

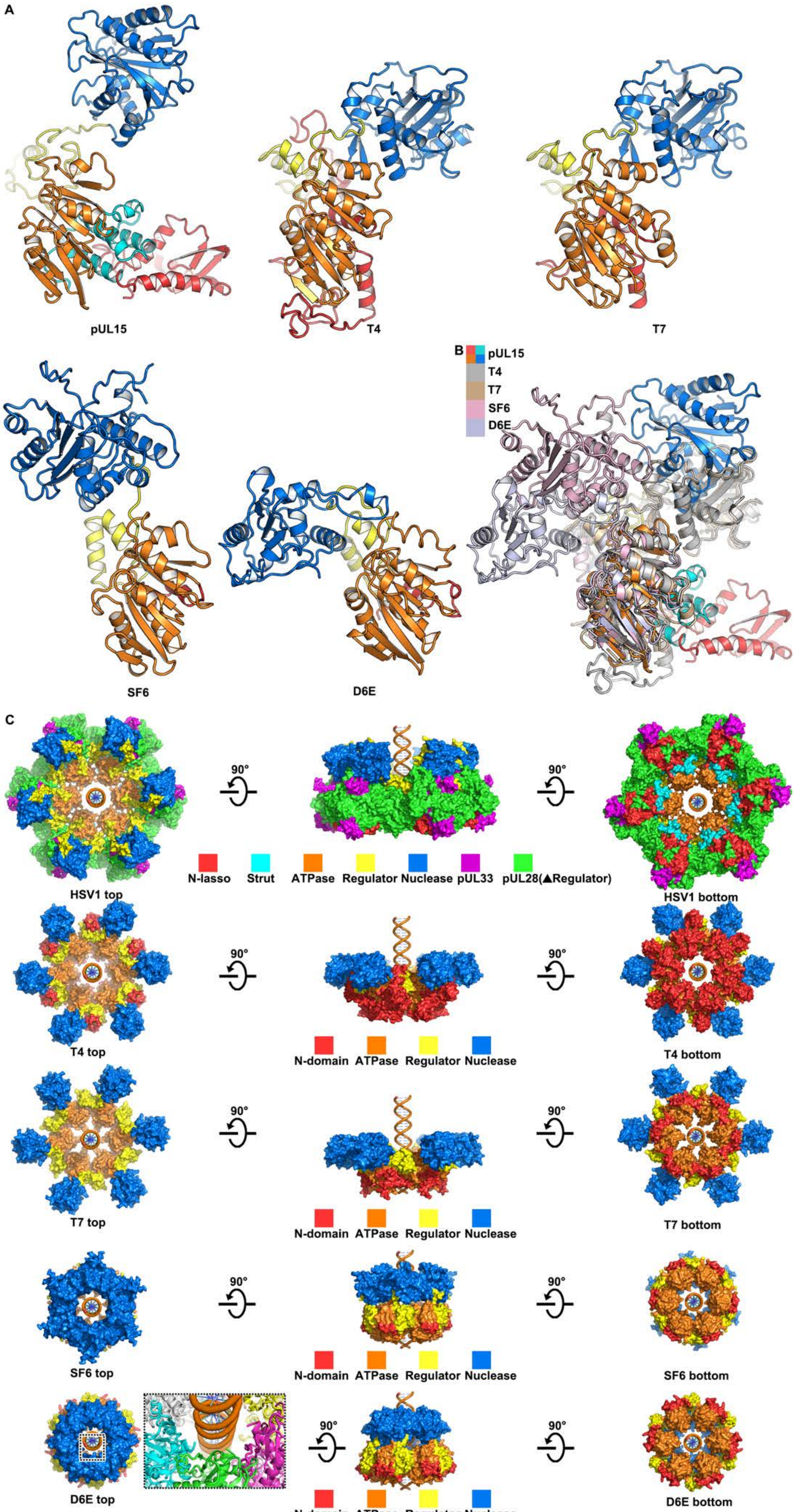

A
pUL15
T4
T7
SF6
D6E
B
pUL15
T4
T7
SF6
D6E
C
90°
N-lasso
Strut
ATPase
Regulator
Nuclease
pUL33
pUL28(▲Regulator)
HSV1 top
HSV1 bottom
T4 top
T4 bottom
N-domain
ATPase
Regulator
Nuclease
T7 top
T7 bottom
SF6 top
SF6 bottom
D6E top
D6E bottom

Fig. S10. Domain arrangements of the nuclease and potential determinants for transition between DNA translocation and DNA cleavage. (**A**) Structural comparisons of pUL15 with its four homologs in bacteriophages including T4 (PDB: 3CPE) (Sun et al., 2007), T7 (PDB:4BIL) (Dauden et al., 2013), SF6 (PDB:4IEE) (Zhao et al., 2013) and D6E (PDB:5OE8) (Xu et al., 2017) reveal a flexible domain arrangement for the nuclease. Color scheme is the same as Fig. 2b. (**B**) Superimpositions of pUL15 with the terL structures of T4, T7, SF6 and D6E. (**C**) Models of the terL (T4, T7, SF6 and D6E) ring derived from HSV-1 hexameric terminase assembly. The dsDNA is positioned at the center of the channel. Hexameric models of HSV-1, T4 and T7 might represent the DNA translocation mode and hexameric models of SF6 and D6E possibly indicate a mode for DNA cleavage. Note: severe clashes between adjacent subunits are observed in D6E ring model. With a slightly bigger molecular weight than the ATPase, it seems impossible for the nuclease domains to arrange into a ring with a smaller inner diameter than that formed by ATPase domains due to the severe clashes from neighboring subunits.

Table S1. CryoEM imaging, data processing and refinement statistics

	Apo-hexamer	Apo-blocked	ADP•BeF3-hexamer	ADP•BeF3-blocked
**Data collection and processing**				
Voltage (kV)	300			
Total dose (e-/Å^2)	60			
Defocus range (µm)	1.5-2.5			
Pixel size (Å)	1.04			
Symmetry imposed	C6	C1	C6	C1
Final particles (no.)	43,188	386,700	9,982	42,053
FSC threshold	0.143			
Map resolution (Å)	3.9	3.5	4.4	3.6
**Refinement**				
Initial model used (PDB code)	N/A			
Model composition				
Non-hydrogen atoms	68,664	11,444	68,862	11,477
Protein residues	8,838	1,473	8,838	1,473
Ligands	Zn		Zn, ADP•BeF3	
R.m.s deviations	N/A		N/A	
Bonds lengths (Å)	0.008	0.009	0.006	0.004
Bonds angles (º)	1.12	1.17	0.82	0.71
Validation				
Clash score	7	16	10	15
Poor rotamers (%)	0.4	0.4	0.8	0.8
Ramachandran plot				
Favored (%)	90.78	90.83	90.12	90.22
Allowed (%)	8.40	8.36	8.76	8.61
Disallowed (%)	0.82	0.81	1.12	1.17

Reference:

Dauden, M.I., Martin-Benito, J., Sanchez-Ferrero, J.C., Pulido-Cid, M., Valpuesta, J.M., and Carrascosa, J.L. (2013). Large Terminase Conformational Change Induced by Connector Binding in Bacteriophage T7. Journal of Biological Chemistry *288*, 16998-17007.

Gouet, P., Courcelle, E., and Stuart, D.I. (1999). ESPript: analysis of multiple sequence alignments in PostScript. Bioinformatics *15*, 305-308.

Kucukelbir, A., Sigworth, F.J., and Tagare, H.D. (2014). Quantifying the local resolution of cryo-EM density maps. Nature methods *11*, 63-65.

Sun, S.Y., Kondabagil, K., Gentz, P.M., Rossmann, M.G., and Rao, V.B. (2007). The structure of the ATPase that powers DNA packaging into bacteriophage t4 procapsids. Molecular cell *25*, 943-949.

Xu, R.G., Jenkins, H.T., Antson, A.A., and Greive, S.J. (2017). Structure of the large terminase from a hyperthermophilic virus reveals a unique mechanism for oligomerization and ATP hydrolysis. Nucleic acids research *45*.

Zhao, H., Christensen, T.E., Kamau, Y.N., and Tang, L. (2013). Structures of the phage Sf6 large terminase provide new insights into DNA translocation and cleavage. Proc Natl Acad Sci U S A *110*, 8075-8080.
